# What is more effective: a daily or a weekly formative test?

**DOI:** 10.1007/s40037-015-0178-8

**Published:** 2015-03-31

**Authors:** Leonieke N. Palmen, Marc A.T.M. Vorstenbosch, Esther Tanck, Jan G.M. Kooloos

**Affiliations:** 1 Department of Anatomy, Radboud University Medical Centre Nijmegen, PO Box 9101, 6500 Nijmegen, the Netherlands; 2 Orthopaedic Research Laboratory, Radboud University Medical Centre, Nijmegen, the Netherlands

**Keywords:** Formative test, Student participation, Student satisfaction

## Abstract

**Background:**

Exams in anatomy courses are traditionally summative. Formative testing induces retrieval practice, provides feedback and enhances learning results. We investigated the optimal frequency for retrieval practice during an anatomy course.

**Method:**

During a first-year course, students were offered four online daily quizzes a week that assessed thoracic anatomy. Once a week they received a quiz about abdominal anatomy. Students immediately received feedback afterwards. In the fourth course week, a survey about participation and satisfaction was taken. 424 students participated in the final summative exam. Trunk wall questions were used as a control. Relationship between participation and test results was investigated with a one-way ANOVA.

**Results:**

More frequent participation in formative tests was correlated to higher scores in the summative exam with no difference between daily and weekly quizzes. This effect was found for thorax-abdomen and ‘control’ trunk wall questions. Participation in weekly quizzes was higher (*p* < 0.001). All survey responses showed a significant difference in favour of the weekly quiz (*p* < 0.001).

**Discussion and conclusion:**

Participation in formative quizzes was correlated to summative exam scores. This correlation was not specific for the material tested, probably because of diligence. Student participation and preference were much higher in weekly quizzes.

## Introduction

Anatomy has always been one of the basic sciences of medical school. Anatomical knowledge is traditionally tested by means of summative examination, providing information for pass/fail decisions. Karpicke and Roediger showed that repeated testing produced a large positive effect on long-term retention, while repeated studying had no effect [1]. This is called the ‘testing effect’ [2, 3]. In spaced education this testing effect or retrieval practice is combined with the ‘spacing effect’ [4]. If information is repeated over spaced intervals of time, the retention is better than if information is offered all at once. Kerfoot demonstrated that spaced education also improved retention of medical knowledge [5]. The goal of formative testing is to enhance learning, whereas summative testing is aimed at grading or decision making. Formative testing can also enhance learning through other ways, for instance through improving motivation and study strategy. Following frequent testing, most students increase their study time and change their strategies [4]. Frequent testing also encourages students to space their study efforts [2].

Rolfe [6] proposed that a formative assessment should be voluntary in nature, non-judgmental, available with rapid feedback, and designed with opportunities to remediate deficiencies. Formative assessments should be offered to the students during the learning period [6].According to Chan et al. [7] retrieval practice could also increase retention of related information, i.e. information that was not part of the test itself.Providing feedback after a retrieval attempt supports future successful retrieval [8].

There are indications that formative assessment during a course improves the student’s performance on the final summative exam [9]. Kibble found that students who took formative online quizzes during the course showed better outcomes on summative exams compared with students who chose not to take quizzes. He also showed that offering course credits for taking formative tests increased student participation [10]. Logan et al. [11] described an experiment during a first-year anatomy course in which students could voluntarily take course-content-related quizzes. Although the students were not enthusiastic about the quizzes, a significant improvement on the final exam was found in the scores of those students who actually took the quizzes.

According to Roediger, repeated retrievals outperform single retrievals [2].It is suggested that the best retrieval schedules involve wide spacing of retrieval attempts [12]. Two strategies of retrieval schedules are described. In an equally spaced schedule, retrievals are evenly spaced with the same amount of time between each retrieval [13]. In an expanding schedule, which was first proposed by Landauer and Bjork [14], each retrieval attempt occurs after an increasingly longer interval. Some studies showed no differences in learning effect between these two retrieval schedules (Balota [15] and Logan [16]), while yet another study (Karpicke [17]) concluded that equally spaced practice led to better long-term retention. Karpicke also pointed out that feedback enriched retention in both the expanding and equally spaced conditions. Nevertheless, the general opinion on what is supposed to be the best retrieval schedule to offer formative assessments to students remains unclear.

The aim of the present study was to further investigate the optimal frequency for retrieval practice through formative assessments in educational practice. The research question was: What is the difference between daily and weekly quizzes, offered during an anatomy course, in terms of learning gain, student participation, and student satisfaction?

## Methods

### Ethical considerations

During the introductory lecture on the first day of the course, the students were informed about the procedures and the purpose of the experiment with the daily and weekly quizzes. Later, the students also received this information by email. It was explained to the students that participation in the experiment was voluntary and that students who did not participate still had access to the quizzes. Because of this, participation would not introduce inequality between students. Moreover, the within-subject design guaranteed that no inequality was introduced amongst participants. After merging data from quiz records and assessment results, data were anonymized for analysis and storage. Results presented in this paper cannot be traced back to individual participants. At the end of the course, the participants were asked to give their written consent to use of their records for this study.

### Educational setting

Radboud university medical center, the Netherlands, offers a six-year medical educational programme. In a first-year course, the students are introduced to the gross anatomy of the thorax, abdomen, and pelvis. It is a four-week, 160 h course in which the organs and walls of the thorax, abdomen, and pelvis are covered. Instructional methods used are lectures, self-study assignments, interactive lectures, computer-assisted learning, collaborative learning, laboratory sessions with prosected specimens, and body painting. The students are assessed in a final summative exam at the end of the fourth week.

### The formative tests

Two sets of formative exams were completed by three anatomy teachers. The questions were designed to resemble the content, format and difficulty of the final exam at the end of the course.

**Fig. 1:**
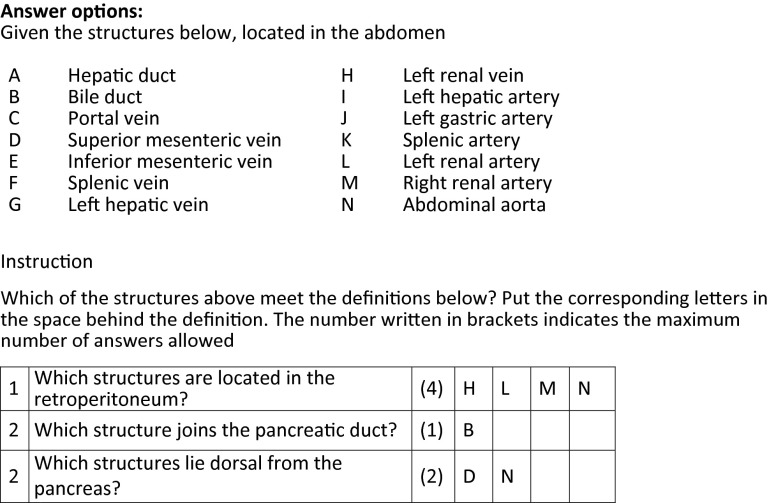
Example of questions in a formative test

During the first three weeks of the course, short daily quizzes about thoracic anatomy were offered to the students on Monday through Thursday. Every quiz had two or three short questions. On Friday a weekly quiz about the abdominal anatomy was offered, containing about ten questions. The daily quizzes and weekly quizzes both consisted of extended matching questions (Fig. 1). The number of questions was similar for daily and weekly quizzes. After a quiz had been completed, immediate feedback was given by showing the right answers. Both forms of formative testing were presented online via Blackboard, the institution’s online education platform. The students were alerted by email when a quiz became available. While a quiz was online, the students could take it as many times as they liked. Availability was limited to discourage saving all the quizzes until later in the course. Each daily quiz was available for 24 h and each weekly quiz for 3.5 days. Participation was registered in Blackboard. Participants received no extra study credits.

### The survey and summative examination

During the fourth week of the course, students were asked to complete a written survey about both forms of formative testing, focusing on their participation and satisfaction. Before we conducted the survey, we optimized its questions with anatomy colleagues. We did not conduct a pilot survey on students.

Three anatomy teachers divided the questions of the summative exam into three categories: thoracic anatomy, abdominal anatomy, and the trunk wall. This last category served as control, because the students did not take quizzes on this subject during the course. If there were doubts about the category a question best fitted in, then that question was excluded. Embryological questions were also excluded.

### Adapted Angoff procedure

To compare the scores in the three categories, we needed an indication of the difficulty of the questions used in the summative exam. We therefore used an adapted Angoff procedure [18]. Five senior medical students who assisted teaching during the course estimated the difficulty of every question in the summative exam on a six-point Likert scale (1 = very simple; 6 = very difficult). These difficulty ratings were averaged across the raters for every question and then multiplied with the student scores on the question to obtain a score that was corrected for difficulty. These five students were blinded for the purpose of this procedure.

### Outcome measures and data analysis

Students were included in this study if they participated in the summative exam and gave written consent to this investigation by completing the survey. The total score on the summative exam was registered and divided into scores on thoracic anatomy, abdominal anatomy, and body wall anatomy. These scores were mathematically corrected for difficulty by using the results of the adapted Angoff procedure.

Participation in daily quizzes was divided into quartiles: 0–3 quizzes completed [0], 4–6 quizzes completed [1], 7–9 quizzes completed [2], 10–12 quizzes completed [3]. Participation in weekly quizzes was divided in quartiles as well: 0 quizzes completed [0], 1 quiz completed [1], 2 quizzes completed [2], 3 quizzes completed [3].

The relationship between participation in both forms of formative testing and the scores of the students in the summative exam were analyzed with a one-way ANOVA.

The students’ opinions about the quizzes were enquired about in a survey (Table 1). This survey was analyzed by means of a paired samples t-test.

**Table 1:** Student survey

Question		Mean	Paired sample t-test
I always participated in the quizzes	Daily quiz	3.56	t = 13.52 df = 423 *p* = 0.000
	Weekly quiz	2.67	
I perceived doing the quizzes as useful	Daily quiz	2.42	t = 7.55 df = 410 *p* = 0.000
	Weekly quiz	1.99	
The quizzes were available long enough	Daily quiz	3.70	t = 13.95 df = 416 *p* = 0.000
	Weekly quiz	2.57	
^a^How many times did I take the quizzes?	Daily quiz	1.186	t = −1.94 df = 418 *p* = 0.053
	Weekly quiz	1.25	
The quizzes enhanced my study efforts	Daily quiz	4.10	t = 7.77 df = 416 *p* = 0.000
	Weekly quiz	3.71	
Other courses should offer comparable quizzes	Daily quiz	2.20	t = 7.55 df = 418 *p* = 0.000
	Weekly quiz	1.84	
I would take quizzes like this in other courses	Daily quiz	2.31	T = 8.40 Df = 418 *p* = .000
	Weekly quiz	1.94	

The questions were answered using a six-point Likert scale (1 = totally agree, 6 = totally disagree)

^a^The students filled in how many times they completed the quizzes (1 = 1 time, 5 = 5 + times)

## Results

### Student participation rates

#### Daily quizzes

A total of twelve daily quizzes were offered to the students during the course period. Quiz participation ranged from 0 to 12 quizzes. Participation in daily quizzes was as follows: 39.2 % completed 0–3 quizzes, 18.6 % 4–6 quizzes, 7–9 quizzes were completed by 20 % of the students and 22.2 % completed 10–12 quizzes. Closer to the summative exam, a significantly higher participation was found. During the first week the average participation rate was 1.58 quizzes versus 2.06 quizzes in the third week (repeated measures ANOVA *F* = 34.4; *p* < 0.001).

#### Weekly quizzes

Regarding the weekly quizzes, participation ranged from 0 to 3 quizzes. Sixty-three students (14.9 %) did not complete any of the quizzes. A total of 12.3 % of the students completed one weekly quiz, while 22.9 % completed two quizzes. Half of the students (50 %) completed all three weekly quizzes. Participation rate in the third week of the course period was significantly higher than participation rate in the first week. The average participation rate during the first week was 0.65 versus 0.76 during the third week (repeated measures ANOVA *F* = 19.7; *p* < 0.001). This rate is below 1.0 because only one weekly quiz per week was offered to the students.

### Effect of quiz participation on the summative exam

Positive relationships were found between the number of quizzes that the students completed (participation rate) and their scores in the final summative exam. This relationship did not change when the scores in the summative exam were corrected for difficulty and was observed for both the daily (Table 2) and the weekly quizzes (Table 3). Scores for questions about the thorax and abdomen as well as scores for questions about the trunk wall (Table 2 and 3) significantly increased with participation rate in both forms of formative quizzes. This shows there was no difference between daily and weekly quizzes with respect to their effect on learning.

**Table 2:** Cross-tabulation of participation rate in the daily quizzes versus total and partial summative test scores presented as a percentage of maximal score and corrected for difficulty

Participation rate	Total score summative test (%)	Score thorax (%)	Score abdomen (%)	Score trunk wall (%)
0–3	70.8	74.2	70.0	69.3
4–6	73.9	76.8	74.0	72.1
7–9	76.3	79.3	75.3	75.2
10–12	82.6	83.1	77.0	79.1
One-way ANOVA	df = 3/423 F = 24.06 *p* = 0.000	df = 3/423 F = 15.65 *p* = 0.000	df = 3/423 F = 12,27 *p* = 0.000	df = 3/423 F = 17.22 *p* = 0.000

**Table 3:** Cross-tabulation of participation rate in the weekly quizzes versus total and partial summative test scores, presented as a percentage of maximal score and corrected for difficulty

Participation rate	Total score summative test (%)	Score thorax (%)	Score abdomen (%)	Score trunk wall (%)
0	69.1	72.3	68.2	67.9
1	70.8	74.4	70.7	68.7
2	73.5	77.1	72.6	72.0
3	77.3	80.3	75.9	76.4
One-way ANOVA	df = 3/423 F = 20.55 *p* = 0.000	df = 3/423 F = 11.81 *p* = 0.000	df = 3/423 F = 12.65 *p* = 0.000	df = 3/423 F = 14.32 *p* = 0.000

### Student survey

A total of 424 students consented to participate in the experiment and filled in the survey about their quiz experience (Table 1). All questions showed a significant difference in favour of the weekly quizzes (*p* < 0.001), except the question about how many times they did each quiz (*p* = 0.053). Students were honest in the survey about their participation: reported participation and registered participation were pretty similar. The students indicated that both the daily and the weekly quizzes did not enhance their study efforts. The weekly quizzes were strongly preferred by 42.5 % of the students versus 12.3 % of the students who preferred the daily quizzes. They also stated they had learned more from the weekly quizzes (48 vs. 6.8 %). If they had to choose between the two forms of formative testing, 60.5 % of the students had a strong preference for the weekly quiz. The daily quiz was preferred by 11.1 % of the students.

## Discussion

Comparing daily and weekly quizzes we found no effect on learning gain. However, a higher student participation for weekly quizzes was found as was a higher preference for these.

### Learning gain

The results of this study showed that more frequent participation in both forms of formative tests is correlated to higher scores on all three discerned parts of the summative exam and this suggests a causal relationship. Results did not show a difference between the anatomical categories that were enquired about in daily quizzes on one hand and in weekly quizzes on the other. Moreover, even the scores in the control category (trunk wall) increased with the participation in both daily quizzes and weekly quizzes. These findings indicate that no difference exists between the influence of the daily and weekly quizzes on learning.

Diligence may be an important confounder. Diligence may be described as the effort expended by students to achieve [19]. Honea found a statistically significant relationship between student diligence and academic achievement [20]. More diligent students are more likely to participate in formative testing and also more likely to perform better on the summative examination. Students who exploit all learning possibilities, including quizzes like in this study, have a higher chance of successful outcomes [21]. To obtain an indication of the diligence of the individual student and to correct for this diligence in future research, we could possibly correlate test results of other courses to our test results. We could also use one of the reliable and valid instruments developed to measure student’s diligence, for example the Diligence Inventory-Higher Education (DI-HE), designed by Bernard and Schuttenberg [22].

We did not determine the influence of gender on our results, but gender might also be a confounder. Previous research has shown that women perform better than men [23, 24], but recent findings by Masui et al. [25] show that gender only occasionally plays a role in achievement. On the other hand, Masui et al. show in the same study that feminine gender positively influences study time and that more study time predicts better grades.

Furthermore, all other learning activities in our educational setting (lectures, self-study assignments, interactive lectures and so on) might have subdued the testing effect that was found previously by Roediger in a non-educational setting [2]. Our study did not register how students studied the course content.

### Student participation

Participation rate in the weekly quizzes was much higher compared with the participation rate in the daily quizzes. Comments from the survey showed that students were annoyed by the fact they received a daily quiz accompanied by an email every day. Contrary to the scattered contents of the daily quiz, weekly quizzes provided some kind of overview of the work done that week. Moreover, the weekly quiz was available longer online than the daily quiz and also available during the weekend. Participation in the third week of the course was higher than in the first week, probably because of the upcoming exam.

### Student satisfaction

The results of the survey showed that students perceived both the daily and weekly quizzes as useful. This is probably because of the voluntary basis and the immediate feedback after completing the quizzes. Student reactions in other studies confirm this [12, 26].

In the student survey, 88 students (20.8 %) stated that the quizzes should be online for a longer period of time. It is understandable that quizzes available at any time would be more popular than quizzes, only available for 24 h, like the daily quizzes. However, to obtain objective data during this study, it was necessary to give the quizzes under controlled conditions. A small number of 15 students (3.5 %) felt the questions about the thoracic anatomy were too easy; they had expected growing difficulty of these questions during the course.

## Conclusion

Participation of students in formative quizzes in an anatomy course is correlated to the scores on the final summative exam. We found no difference in effect on test results between daily and weekly quizzes. Based on the higher student participation and student satisfaction we propose weekly quizzes as a more successful retrieval schedule than daily quizzes.

## Essentials

- Formative testing induces retrieval practice, provides feedback and enhances learning results.
- In this study, we investigated the optimal frequency for retrieval practice during an anatomy course by offering both daily and weekly quizzes to the students.
- The effect of daily and weekly quizzes on test results, student participation and student satisfaction was studied.
- Participation of students in formative quizzes in an anatomy course is correlated to test scores, but it is not specific for the test material, probably because of diligence.
- Student participation and preference are much higher for weekly quizzes.
